# Kinetic Analysis of Zinc/Cadmium Reciprocal Competitions Suggests a Possible Zn-Insensitive Pathway for Root-to-Shoot Cadmium Translocation in Rice

**DOI:** 10.1186/s12284-016-0088-3

**Published:** 2016-04-12

**Authors:** Laura Fontanili, Clarissa Lancilli, Nobuo Suzui, Bianca Dendena, Yong-Gen Yin, Alessandro Ferri, Satomi Ishii, Naoki Kawachi, Giorgio Lucchini, Shu Fujimaki, Gian Attilio Sacchi, Fabio Francesco Nocito

**Affiliations:** Dipartimento di Scienze Agrarie e Ambientali - Produzione, Territorio, Agroenergia (DISAA), Università degli Studi di Milano, 20133 Milan, Italy; Istituto di Biologia e Biotecnologia Agraria (IBBA), Consiglio Nazionale delle Ricerche, 20133 Milan, Italy; Department of Radiation-Applied Biology, Takasaki Advanced Radiation Research Institute, Quantum Beam Science Research Directorate, National Institutes for Quantum and Radiological Science and Technology (QST), 1233 Watanuki, Takasaki, Gunma 370-1292 Japan

**Keywords:** Rice, Cadmium, Zinc, Translocation, PETIS

## Abstract

**Background:**

Among cereals, rice has a genetic propensity to accumulate high levels of cadmium (Cd) in grains. Xylem-mediated root-to-shoot translocation rather than root uptake has been suggested as the main physiological factor accounting for the genotypic variation observed in Cd accumulation in shoots and grains. Several evidence indicate OsHMA2 – a putative zinc (Zn) transporter – as the main candidate protein that could be involved in mediating Cd- and Zn-xylem loading in rice. However, the specific interactions between Zn and Cd in rice often appear anomalous if compared to those observed in other staple crops, suggesting that root-to-shoot Cd translocation process could be more complex than previously thought. In this study we performed a complete set of competition experiments with Zn and Cd in order to analyze their possible interactions and reciprocal effects at the root-to-shoot translocation level.

**Results:**

The competition analysis revealed the lack of a full reciprocity when considering the effect of Cd on Zn accumulation, and vice versa, since the accumulation of Zn in the shoots was progressively inhibited by Cd increases, whereas that of Cd was only partially impaired by Zn. Such behaviors were probably dependent on Cd-xylem loading mechanisms, as suggested by: i) the analysis of Zn and Cd content in the xylem sap performed in relation to the concentration of the two metals in the mobile fractions of the roots; ii) the analysis of the systemic movement of ^107^Cd in short term experiments performed using a positron-emitting tracer imaging system (PETIS).

**Conclusions:**

Our results suggest that at least two pathways may mediate root-to-shoot Cd translocation in rice. The former could involve OsHMA2 as Zn^2+^/Cd^2+^ xylem loader, whereas the latter appears to involve a Zn-insensitive system that still needs to be identified.

**Electronic supplementary material:**

The online version of this article (doi:10.1186/s12284-016-0088-3) contains supplementary material, which is available to authorized users.

## Background

Cadmium occurs naturally in the environment as a result of volcanic emissions and the weathering of rocks. Anthropogenic activities can contribute to increase the background levels of Cd in soil, water and living organisms. Important sources of soil contamination are short- or long-range atmospheric depositions from mining activities, phosphate fertilizers and manures, municipal sewage wastes, urban composts and industrial sludges (Alloway and Steinnes 1999; McLaughlin et al. 1999).

Plant-derived foods are the major source of Cd exposure for humans (EFSA 2009). Although Cd is not essential for plant nutrition, it can be easily taken up from the soil by plant roots and accumulated in different plant organs. In this way, Cd contamination of soils can result in moderate Cd accumulation in the edible parts of staple crops, which, in turn, represent the major pathway for Cd entry into the agricultural food chain, as well as the major route for chronic Cd exposure in general populations (Wagner 1993; FAO/WHO 2001). In humans, Cd is known to accumulate in the kidneys and shows a long biological half-life in the whole body, ranging from 10 to 33 years (Ellis et al. 1979).

Among cereals, rice (*Oryza sativa* L.) has a genetic propensity to accumulate high level of Cd in grains (Arao and Ae 2003), sometimes reaching values exceeding the limit of 0.4 mg kg^−1^ established by the Codex Alimentarius Commission of FAO/WHO (CODEX STAN 193–1995 2015). Interestingly, earlier investigations reported finding that rice-eating ethnic groups such as the people from Japan, Thailand, Hong Kong and Taiwan, have the highest renal Cd levels in the world (Perry et al. 1961) and that long-term consumption of rice grown in Cd-contaminated paddy soils can cause a high prevalence of renal proximal tubular dysfunction (Kobayashi 1978; Cai et al. 1990; Kobayashi et al. 2002).

Since rice is the staple crop of more than half of the world’s population, most of the Cd exposure through food is likely to come from the consumption of this cereal (Clemens et al. 2013a). For this reason, several efforts have been made to develop a number of agronomic techniques aimed at minimizing the absorption of Cd by rice (Murakami et al. 2007; Makino et al. 2008; Arao et al. 2010) as well as to understand both the biological bases and the genetic variation of Cd accumulation in rice grains, with the special aim of developing new low-Cd varieties (Clemens et al. 2013a; Uraguchi and Fujiwara 2013). This last approach seems to be particularly promising since the existence of a high variability for the trait “Cd accumulation in grain” represents a good starting point for the new challenges of plant breeding (Arao and Ae 2003; Grant et al. 2008; Shi et al. 2009; Uraguchi et al. 2009).

Several processes have been described as involved in Cd accumulation in rice grains. Briefly, Cd is absorbed by roots via transport pathways normally involved in the acquisition of mineral nutrients. OsNramp5 (natural resistance-associated macrophage protein 5) – a Mn transporter – has recently been described as the major Cd uptake system in rice (Ishikawa et al. 2012; Sasaki et al. 2012), although Zn and Fe transporters with limited specificity – such as OsZip1 (Zrt/Irt-like protein 1), OsIRT1 and OsIRT2 (iron regulated transporter 1 and 2), and OsNramp1 – may be implicated in Cd uptake under Zn or Fe deficiency (Ramesh et al. 2003; Nakanishi et al. 2006; Takahashi et al. 2011). Once inside root cells, Cd is translocated to the shoot through the xylem, and then accumulated in leaves and stems. Finally, during the reproductive stage phloem transport seems to be the main process mediating Cd accumulation in the grains (Tanaka et al. 2007; Kato et al. 2010). During this phase Cd remobilization from leaves as well as xylem-to-phloem Cd transfer in the nodes are thought to be essential to determine the final level of Cd into the grains (Rodda et al. 2011; Fujimaki et al. 2010; Uraguchi et al. 2011; Yamaguchi et al. 2012; Kobayashi et al. 2013). Among all these processes xylem-mediated root-to-shoot translocation rather than root uptake has been suggested as the main and most common physiological factor accounting for the genotypic variation observed in Cd accumulation in shoots and grains of rice plants (Uraguchi et al. 2009; Ishikawa et al. 2011). Translocation, in turn, mainly results from the equilibrium between the capability of each rice genotype to retain Cd in the root and the activities involved in xylem loading (Ueno et al. 2010; Miyadate et al. 2011; Nocito et al. 2011). In this equilibrium, a complex root firewall system – involving the vacuolar sequestration of free Cd ions (Ueno et al. 2010; Miyadate et al. 2011) or Cd-phytochelatin complexes (Nocito et al. 2011) – traps the excess of Cd within the root, determining, in this way, the total amount of Cd ions potentially available to be translocated via the xylem in a root-to-shoot direction.

Translocation of Cd ions to the shoots requires active loading into the xylem (Colangelo and Guerinot 2006). Several experimental sources of evidence indicate OsHMA2 – a putative Zn transporter belonging to the P_1B_-type ATPase family – as the main candidate protein that could be involved in mediating Cd- and Zn-xylem loading in rice (Nocito et al. 2011; Satoh-Nagasawa et al. 2012; Takahashi et al. 2012). On the other hand, Yamaji et al. (2013) proposed OsHMA2 as an influx transporter for both Cd and Zn, involved in the preferential distribution of the two metal ions through the phloem to the developing tissues.

OsHMA2-defective mutants showed a lower translocation ratio for both Zn and Cd and reduced Cd accumulation into the grains as compared to the wild types (Satoh-Nagasawa et al. 2012; Takahashi et al. 2012). Moreover, a careful analysis of mutant phenotypes clearly reveals that root-to-shoot Cd translocation in rice could be a more complex process than previously thought, since that the lack of OsHMA2 activity had more and stronger effects on Zn than on Cd translocation, which indeed was not completely impaired by the mutation (Satoh-Nagasawa et al. 2012). Such a behavior suggests the hypothesis that Cd and Zn may only partially share the same pathways for translocation from root to shoot, underlining at the same time important implication for food safety, especially in the cases where the strategies used for containing Cd accumulation in crops are founded on Zn fertilization (Oliver et al. 1994; Fahad et al. 2015). Considering these aspects, in this paper we present and discuss a set of competition experiments with Zn and Cd which aimed at analyzing their possible interactions and reciprocal effects at the root-to-shoot translocation level.

## Results

To better characterize the possible interactions between Zn and Cd translocation pathways we performed two set of experiments. In the first, rice plants were hydroponically grown and exposed for a 10-day period to a range of three increasing Zn external concentrations (0.1, 1, and 10 μM) in the absence or presence of a steady amount of Cd (0.1 μM); in the second, plants were exposed for 10 days to different Cd concentrations (0, 0.01, 0.1, and 1 μM) in the presence of a steady amount of Zn (1 μM). In all the experiments, changes in metal concentrations did not produce either significant effects on the growth of both roots and shoots, or any apparent symptoms of stress (data not shown): at the end of the exposure period, root and shoot dry weights of a single plant were 0.128 ± 0.004 g and 0.588 ± 0.023 g, respectively.

### Cd and Zn Partitioning Between Root and Shoot

**Fig. 1:**
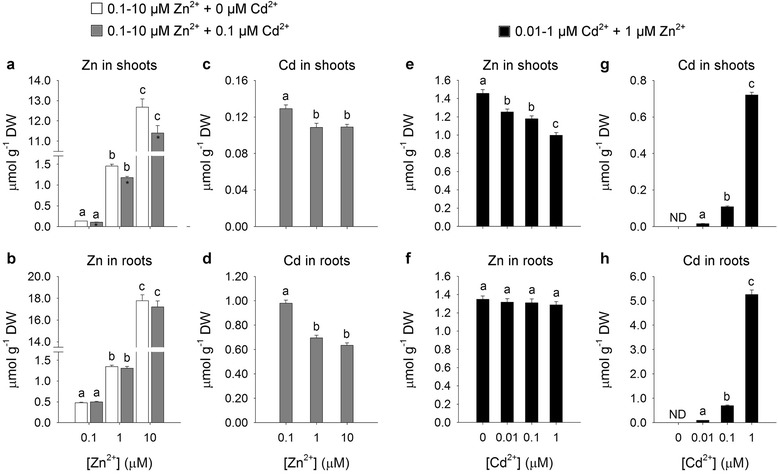
Zn and Cd accumulation in shoots and roots. Rice plants were hydroponically grown and exposed for a 10-day period to increasing Zn external concentrations (from 0.1 to 10 μM), in the absence or presence of 0.1 μM Cd^2+^ (**a**-**d**), or to different Cd concentrations (from 0 to 1 μM) in the presence of 1 μM Zn^2+^ (**e**-**h**). **a** and **b** Zn concentration in shoots and roots in the absence (white bars) and in the presence (grey bars) of a steady amount of Cd. **c** and **d** Cd concentration in shoots and roots in the presence of a steady amount of Cd. E and F, Zn concentration in shoots and roots in the presence of a steady amount of Zn. **g** and **h** Cd concentration in shoots and roots in the presence of a steady amount of Zn. Bars and error bars are means and SE of three experiments run in triplicate (*n* = 9). Different letters indicate significant differences between treatments (*P* < 0.05). Asterisks indicate significant differences between plants exposed or not to 0.1 μM Cd^2+^ (*P* ≤ 0.001). ND, not detectable; DW, dry weight

Results of the first experiment indicated that, in the absence of any source of Cd, Zn concentration in roots and shoots significantly increased as Zn availability in the external medium did. A similar trend was observed in the presence of a steady amount of Cd (0.1 μM); however, in this condition, Zn concentration in the shoots was lowered by the presence of Cd, whilst in the roots it was not significantly affected by the presence of the interfering metal (Fig. 1a and b). Finally, Cd accumulation in shoots and roots was significantly affected changing the external Zn concentration from 0.1 to 1 μM. Interestingly, a further increase in Zn availability in the medium - up to 10 μM - did not produced any additional decrease in Cd accumulation (Fig. 1c and d).

In the second experiment, the concentration of Zn in the shoots significantly decreased as Cd availability in the external medium increased, whilst Zn accumulation in the roots did not seem significantly affected by Cd availability (Fig. 1e and f). Under the same conditions, a dramatic increase in Cd accumulation was observed in both shoots and roots by changing the external Cd concentration from 0.01 to 1 μM (Fig. 1g and h).

### Analysis of Root-to-Shoot Translocation of Zn and Cd

Dynamics of root-to-shoot translocation of Zn and Cd were examined by measuring their concentrations in the xylem sap of rice plants exposed to the different combinations of the metals. In these experiments, translocation was estimated as the amount of Zn or Cd ions loaded and transported in the xylem sap for 45 min.

**Fig. 2:**
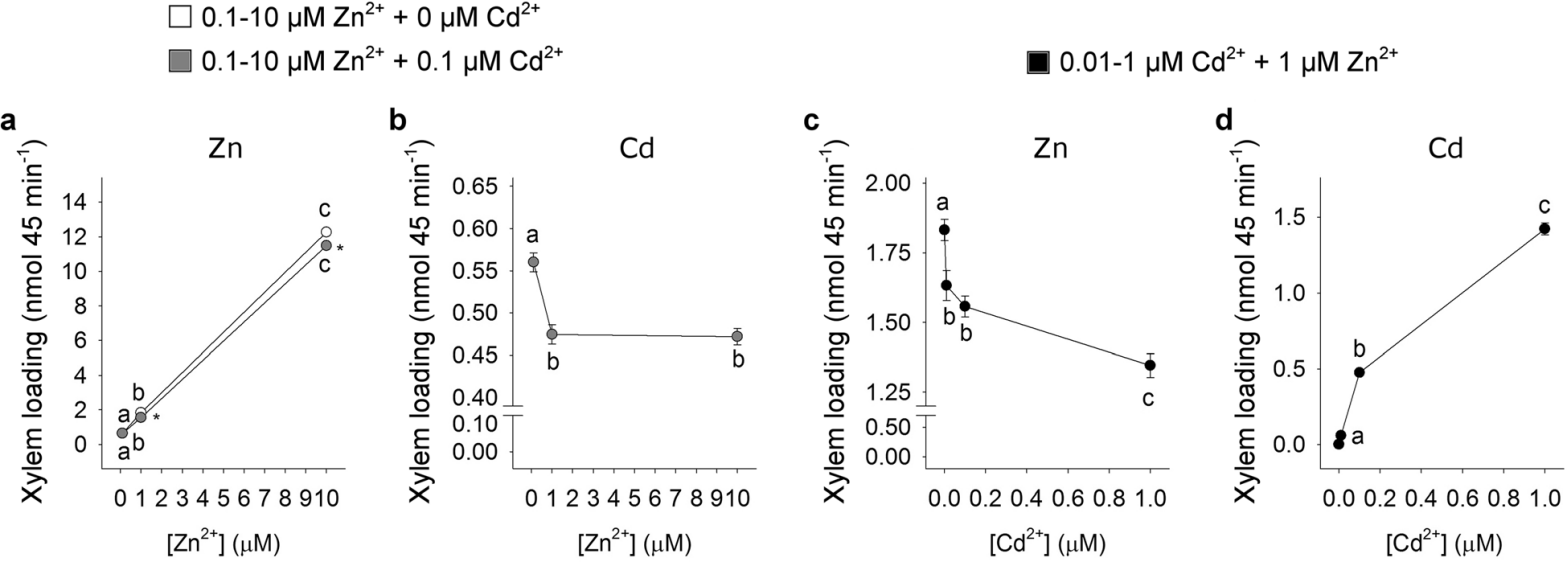
Zn and Cd xylem loading. Rice plants were hydroponically grown and exposed for a 10-day period to increasing Zn external concentrations (from 0.1 to 10 μM), in the absence or presence of 0.1 μM Cd^2+^ (**a** and **b**), or to different Cd concentrations (from 0 to 1 μM) in the presence of 1 μM Zn^2+^ (**c** and **d**). At the end of the exposure period, shoots were separated from roots and the xylem sap exuded from the cut (root side) surface was collected over a 45 min period. **a** Zn ions loaded and transported in the xylem sap in the absence (white circles) and in the presence (grey circles) of a steady amount of Cd. **b** Cd ions loaded and transported in the xylem sap in the presence of a steady amount of Cd. **c** and **d** Zn and Cd ions loaded and transported in the xylem sap in the presence of a steady amount of Zn. Data are means and SE of three experiments run in triplicate (*n* = 9). Different letters indicate significant differences between treatments (*P* < 0.05). Asterisks indicate significant differences between plants exposed or not to 0.1 μM Cd^2+^ (*P* ≤ 0.001)

The amount of Zn loaded in the xylem sap linearly increased as Zn concentration in the external medium did, both in the absence and in the presence of 0.1 μM Cd^2+^ (Fig. 2a). The presence of a steady amount of Cd (0.1 μM) slightly decreased Zn xylem loading in all the conditions analyzed (Fig. 2a). In the same experiment, Cd xylem loading was significantly inhibited by changing the external Zn concentration from 0.1 to 1 μM, and then was not affected by a further increase in Zn availability (Fig. 2b). On the other hand, increases in Cd concentration in the external medium progressively reduced Zn xylem loading (Fig. 2c) and resulted in significant increases in the amount of Cd ions loaded in the xylem sap (Fig. 2d). In the latter case, the Cd xylem loading curve started to approach saturation at 0.1 μM.

In both the experimental setups, the ability to load Zn and Cd into the xylem was linearly related to the total amount of Zn and Cd ions accumulated in the shoots over the 10-day period (Additional file 1: Figure S1).

### Effect of Zn and Cd Exposure on Thiol Biosynthesis

**Fig. 3:**
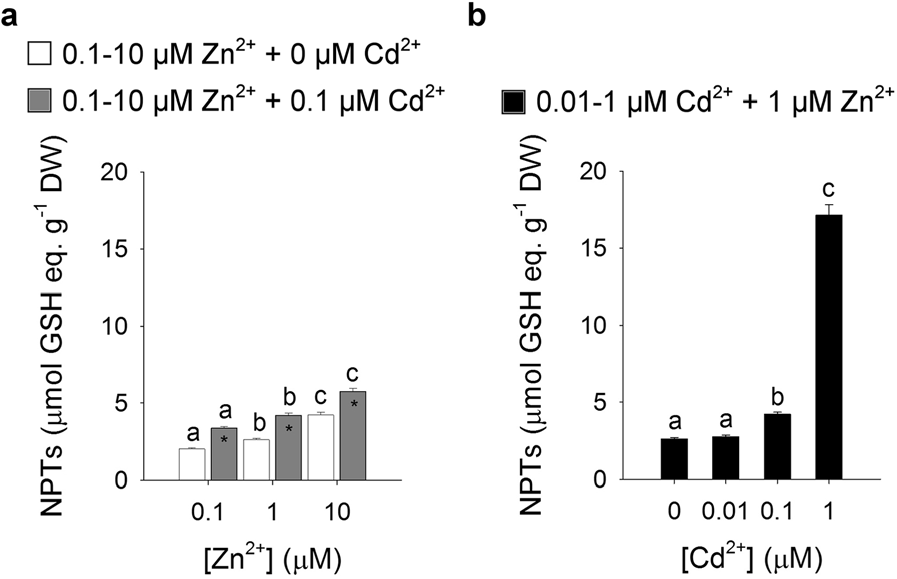
Total non-protein thiols (NPTs) in the roots. **a** Rice plants were exposed for a 10-day period to increasing Zn external concentrations (from 0.1 to 10 μM), in the absence (white bars) or presence (grey bars) of 0.1 μM Cd^2+^. **b** Rice plants were exposed for a 10-day period to different Cd concentrations (from 0 to 1 μM) in the presence of 1 μM Zn^2+^. NPT levels are expressed as GSH equivalents. Bars and error bars are means and SE of three experiments run in triplicate (*n* = 9). Different letters indicate significant differences between treatments (*P* < 0.05). Asterisks indicate significant differences between plants exposed or not to 0.1 μM Cd^2+^ (*P* ≤ 0.001). DW, dry weight

Since activation of thiol metabolism may potentially allow a greater proportion of Zn and Cd to be retained in roots through vacuolar sequestration, we measured the levels of non-protein thiols (NPTs) in the roots of plants exposed to the different combinations of the two metals. The NPT levels of the roots increased as the Zn concentration in the external medium did, either in the absence or in the presence of 0.1 μM Cd^2+^. Interestingly, the levels of NPTs measured for each Zn exposure condition significantly increased with the concomitant presence of Cd in the media (Fig. 3a). Finally, the NPT levels in the roots significantly increased as the external Cd concentration did (Fig. 3b).

### Fractioning of Zn and Cd in Rice Roots

Fractioning of Zn and Cd retained by roots was carried out using a sequential extraction procedure with buffer and acid (Rauser and Meuwly 1995; Nocito et al. 2011). Additional file 1: Tables S1 and S2 summarize results obtained in representative experiments where the sum of Zn and/or Cd ions recovered in the different fractions accounts for at least 97 % of the total Zn and/or Cd content of the roots. Following extraction, three main metal-ion fractions were obtained: i) buffer soluble (extracts 1–6); ii) acid soluble (extracts 7–9); iii) ash (non-soluble Zn and/or Cd). Extracts 1 to 6 were further resolved into two fractions, named anionic and cationic, by anion-exchange chromatography (Additional file 1: Figure S2).

The procedure which we used for metal ion fractioning allowed us to discriminate Zn and Cd ions: i) retained in complexes with thiol-peptides or other soluble molecules negatively charged in the extraction buffer (anionic fraction); ii) in free cytosolic form and/or bound with ligands (i.e. organic acid and nicotianamine) to form cellular complexes of relatively low thermodynamic stability (cationic fraction); iii) tightly adsorbed to cellular matrices or apoplast components (acid soluble and ash). So, only Zn and Cd ions in the cationic fraction should be considered available for root-to-shoot translocation (Nocito et al. 2011).

An integrated analysis of data in which the total amount of each metal ion retained by roots is divided into two fractions – named mobile (cationic, i.e. potentially available for root-to-shoot translocation) and non-mobile (anionic + acid soluble + ash) – according to Nocito et al. (2011) is reported in Table 1. Results indicated that both mobile and non-mobile fractions of Zn were linearly related to the Zn concentration in the external medium, either in the absence or in the presence of Cd; however, the presence of a steady amount of Cd in the medium significantly enhanced the concentration of Zn ions in the mobile fractions because of the lowering in the amount of the metal in the non-mobile fractions. In these last conditions, increasing the Zn external concentration led to decreased Cd content in the roots (Additional file 1: Tables S2) but did not produce any significant effect on the amount of Cd in the mobile fraction (Table 1). On the other hand, increasing the Cd external concentration significantly increased the amount of Zn and Cd ions measured in the mobile fractions (Table 1).

**Table 1:** Partitioning of Zn and Cd ions of the roots between potentially mobile and non-mobile fractions

Metal concentration		Mobile fraction		Non-mobile fraction	
Zn	Cd	Zn	Cd	Zn	Cd
*μM*		*nmol g* ^*−1*^ *DW*			
0.1	0	143.03 ± 3.86 (a)	ND	334.78 ± 11.01 (a)	ND
1	0	420.00 ± 14.70 (a)	ND	927.00 ± 28.54 (a)	ND
10	0	3136.82 ± 128.61 (b)	ND	14636.93 ± 584.18 (b)	ND
0.1	0.1	206.02 ± 6.59 (a)	81.66 ± 2.61 (a)	292.83 ± 10.44 (a)	898.72 ± 32.04 (a)
1	0.1	632.25 ± 22.13 (a)	83.00 ± 2.82 (a)	677.22 ± 26.93 (a)	612.57 ± 20.70 (a)
10	0.1	4541.08 ± 177.10 (b)	82.00 ± 3.53 (a)	12669.39 ± 335.86 (b)	552.86 ± 21.10 (b)
1	0	420.00 ± 14.70 (a)	ND	927 ± 28.54 (a)	ND
1	0.01	486.91 ± 17.04 (a)	11.06 ± 0.32 (a)	829.72 ± 27.26 (a)	80.63 ± 2.76 (a)
1	0.1	632.25 ± 22.13 (b)	83.00 ± 2.82 (b)	677.22 ± 26.93 (b)	612.57 ± 20.70 (b)
1	1	696.00 ± 24.36 (b)	349.49 ± 13.28 (c)	591.15 ± 18.18 (b)	4912.21 ± 137.26 (c)

Rice plants were hydroponically grown and exposed for a 10‐day period to increasing Zn external concentrations (from 0.1 to 10 μM), in the absence or presence of 0.1 μM Cd^2+^, or to different Cd concentrations (from 0 to 1 μM) in the presence of 1 μM Zn^2+^. Mobile (cationic) and non-mobile (anionic + acid soluble + ash) metal ion fractions are derived using data reported in Additional file 1: Table S1 and S2. Data are means and SE of three experiments, each performed with eight plants (*n* = 3). Different letters indicate significant differences between treatments (*P* < 0.05). ND, not detectable; DW, dry weight

Finally, the anionic buffer-soluble fractions were further resolved by gel filtration on a Sephadex G-50 column into peaks I, II and III (Additional file 1: Figure S3). Zn/Cd ions into peak I at the void volume of the column (*V*_*0*_) were ascribed to non-specific adsorption of the metal ions to proteins. Peaks II and III – centered between *V*_*0*_ and *V*_*t*_ of the column – were designated as the classical high-molecular-weight (HMW) thiol based Cd-binding complexes and low-molecular-weight (LMW) thiol based Zn- and/or Cd-binding complexes, respectively, since the amount of NPTs recovered in these fractions accounted for 22 (1 μM Zn^2+^, 0 μM Cd^2+^) to 89 % (1 μM Zn^2+^, 1 μM Cd^2+^) of the total GSH equivalents measured in the roots; no thiols were found in peaks II and III of the anionic buffer-soluble fraction obtained from roots of plants grown under 0.1 μM Zn^2+^ in the absence of Cd^2+^ (data not shown). Neither Zn nor Cd ions were found at total volume (*V*_*t*_) of the column (*K*_*av*_ = 1). Peaks II (HMW) were centered around *K*_*av*_ = 0.44 for Cd, whilst peaks III (LMW) were centered around *K*_*av*_ = 0.58 and *K*_*av*_ = 0.62, for Zn and Cd, respectively. Data analysis revealed that rice roots sequestered Zn and Cd differently. In all cases Zn appeared in LMW complexes. In the absence of any source of Cd, LMW Zn-binding complexes significantly increased as the Zn external concentration did. Interestingly, the presence of Cd in the external medium significantly enhanced the amount of Zn ions found in LMW complexes. Moreover, the amount of Cd ions in LMW complexes remained constant in all the Zn conditions analyzed, differently from that found in HMW complexes, which instead decreased as Zn external concentration increased. On the other hand, LMW and HMW Cd-binding complexes appeared in a dynamic equilibrium depending on Cd external concentrations, as indicated by the ratio between the amount of Cd ions retained in each complex, which notably changed when moving toward the highest Cd external concentration, in the presence of a steady amount of Zn (Additional file 1: Tables S3 and S4). Finally, in the same conditions, the amount of Zn found in LMW complexes significantly increased as Cd external concentration did.

### Kinetic Analysis of ^107^Cd Systemic Movement

**Fig. 4:**
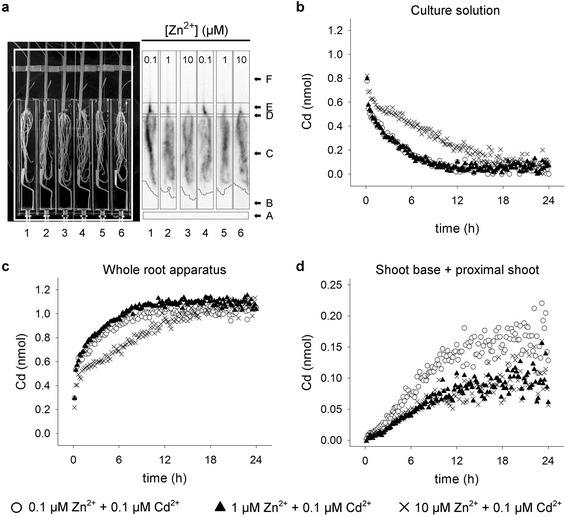
Time-course analysis of Cd systemic movement. Rice plants were exposed for a 24-h period to different Zn concentrations (from 0.1 to 10 μM), in the presence of 0.1 μM Cd^2+^ enriched with ^107^Cd. **a** Image of rice plants used in a typical PETIS experiment. The field of view of PETIS is bordered by the white continuous line. ROIs used for time-course analysis are indicated with arrows in the adjacent panel. ROI-A, background; ROI-B, culture solution; ROI-C, distal root; ROI-D, proximal root; ROI-E, shoot base; ROI-F, proximal shoot. **b** and **d** Time-course analysis of Cd dynamics in the culture solution (**b**), whole root apparatus (**c**), and shoot base + proximal shoot (**d**). White circles, black triangles, and thin x refer to the experiments performed in the presence of 0.1 (No. 4), 1 (No. 5) and 10 (No. 6) μM Zn^2+^, respectively. A representative set of data from two independent experiments performed with two plants for each Zn exposure condition is given

The systemic movement of Cd in the whole plant was further analyzed using ^107^Cd in short-term (24 h) PETIS experiments. Figure 4a shows the field of view of a typical experiment and the regions of interest (ROIs) used to estimate the dynamics of Cd in the plants. In particular, six ROIs (background, culture solution, distal roots, proximal roots, shoot base, and proximal shoot) were set for each plant. The experiments were started by applying to the roots fresh marked (^107^Cd; 0.55 MBq mL^−1^) culture solutions containing 0.5 mM CaCl_2_, 0.1 μM CdCl_2_, and different concentrations of Zn^2+^ (0.1, 1, and 10 μM). ^107^Cd absorption by roots was immediately observed after injection as clearly shown by the comparison of Fig. 4b and c. In all the conditions analyzed, the amount of Cd in the roots increased over time, reaching a common maximum plateau value at about 11 h both for plants exposed to 0.1 and 1 μM Zn^2+^, and about 16 h for plants exposed to 10 μM Zn^2+^ (Fig. 4c). Concerning the shoots (Fig. 4d), ^107^Cd signals appeared in the lower parts of the stems (shoot bases; Additional file 1: Figure S4) within 1 h from the injections and then linearly increased at least up to 10 h. Considering the initial slope of each curve (from 0 to 10 h) we estimated that the rate of Cd translocation was significantly higher in plants exposed to 0.1 μM Zn^2+^ (12.6 ± 0.3 pmol h^−1^) compared with those exposed to 1 or 10 μM Zn^2+^, for which the estimated rates of Cd translocation were similar (7.8 ± 0.2 pmol h^−1^ or 7.5 ± 0.4 pmol h^−1^, respectively). Similar results were obtained in a second independent analysis (Additional file 1: Figure S5).

## Discussion

The specific interactions between Zn and Cd in rice – recently reviewed by Chaney (2015) – often appear anomalous if compared to those observed in other staple crops, since the routes used by Cd to enter root cells seem to be closely dependent on Zn nutritional status. Honma and Hirata (1978) described a noticeable effect of Zn starvation on Cd uptake. On the other hand, under Zn sufficiency or excess Cd uptake and accumulation become independent from the levels of Zn in the soils, in strong contrast with other cereal grains and vegetables (Chaney 2015). The peculiar ability of rice to accumulate soil Cd and not Zn in the grains when grown on Zn/Cd co-contaminated soils, in which the level of Zn are up to 100-fold higher than those of Cd, is impressive (Simmons et al. 2003; Chaney 2010). Such a behavior has important implications for human health in subsistence rice consumers, because the level of dietary Zn are known to influence Cd absorption in animal tissues (Fox et al. 1984; Fox 1988; McKenna et al. 1992; Reeves and Chaney 2004). It is now clear that the biological bases of these anomalies are likely due to the presence of multiple transport systems able to mediate Cd uptake (see also the introduction section), whose prevalence, ion specificity and regulation may strongly affect the interaction between Zn and Cd at different levels. Such a relative complexity may make it difficult to study the relationship existing between Zn and Cd at the root-to-shoot translocation level, since possible competitions could be prevented or masked by the relative expression of the transport systems involved in Zn and/or Cd uptake at the root level.

To try to clarify these aspects, we performed a complete set of competition experiments with Zn and Cd using rice plants grown in a soil-free system, aimed at obtaining a wide range of Zn and Cd concentrations at the root level. To these purposes we chose to grow plants under a non-saturating range of Zn external concentrations, in order to allow variable amounts of Cd to be absorbed at least through two main transport systems with different selectivity: i) OsZip1 (i.e. a Zn transporter up-regulated under Zn deficiency and able to move both Zn and Cd; Ramesh et al. 2003); ii) OsNramp5 (i.e. a Mn transporter able to move Cd, but not Zn; Sasaki et al. 2012). In our experimental conditions the final Zn/Cd concentration ratio into the roots ranged from 0.24 (Zn^2+^ 1 μM; 1 μM Cd^2+^) to 27.11 (Zn^2+^ 10 μM; 0.1 μM Cd^2+^).

The main results we obtained clearly indicate the lack of a full reciprocity when considering the effect of Cd on Zn accumulation, and vice versa, since the accumulation of Zn in the shoots was significantly inhibited by Cd increases in all the analyzed conditions, whereas that of Cd was only partially impaired by Zn increases (Fig. 1). In fact, Cd accumulation in the shoots was reduced by 16 % by changing the external Zn concentration by one order of magnitude (from 0.1 to 1 μM), then remained unaffected by a further increase in Zn availability, from 1 to 10 μM, indicating that Cd accumulation in this range was a Zn-independent process. However, from these data we cannot conclude that the effects of Zn on shoot Cd accumulation necessarily result from mechanisms involved in root-to-shoot Cd translocation, since Cd absorption by plant roots seemed affected by Zn concentration, as indicated by the total amount of Cd in the whole plant, whose value decreased as Zn concentration in the external medium increased (Fig. 1c and d). A similar observation may be done concerning Zn accumulation in the shoots under different Cd concentrations (Fig. 1e and f). Such behaviors could be reasonably due to the range of Zn concentrations we used for the experiments. However, the ability to load Zn and Cd into the xylem was linearly related to total amounts of Zn and Cd ions accumulated in the shoots over the 10-day period (Fig. 2, Additional file 1: Figure S1). This suggests the hypothesis that the differential effect produced by the two metals on Zn and Cd accumulation in the shoot was reasonably due to the existence of at least two translocation pathways with different metal selectivity. It is also important to consider that the total amounts of Zn and Cd ions in the root tissues are often poor indicators of their actual availability for loading into the xylem and then being translocated in a root-to-shoot direction, since, once inside the cells, Zn and Cd ions may be trapped within the root through selective binding sites or molecules with high affinity for the metals or through transfer across a membrane into an intracellular compartment (Clemens 2006; Ueno et al. 2010; Nocito et al. 2011). Moreover, the selective binding of Zn with low-molecular weight ligand, such as nicotianamine (NA), may promote both symplastic movement and long-distance distribution of the metal ion within the plant (Lee et al. 2011; Deinlein et al. 2012; Haydon et al. 2012). Thus, the concentration of Zn and Cd ions potentially mobile in the plants results from several different biochemical and physiological processes involved in metal chelation, compartmentalization and adsorption (Souza and Rauser 2003; Nocito et al. 2011; Sinclair and Krämer 2012; Clemens et al. 2013b; Olsen and Palmgren 2014).

It has been shown that different photosynthetic organisms respond to excess of Zn and Cd ions by producing phytochelatins (PCs), a class of thiol compounds involved in metal chelation and vacuolar sequestration (Grill et al. 1987; Grill et al. 1988; Zenk 1996; Tennstedt et al. 2009; García-García et al. 2014; Song et al. 2014), which may largely contribute to Zn and Cd retention within the root (Tennstedt et al. 2009; Wong and Cobbett 2009). In our conditions, rising Zn or Cd exposure progressively increased, with different efficiency, the level of NPTs in the roots (Fig. 3). Such a finding may be reasonably ascribable to the activation of PC biosynthesis, as revealed by the gel filtration analysis of the anionic fractions in which most of the Zn and Cd ions were immobilized with thiol compounds in LMW and HMW complexes (Additional file 1: Figure S3, Tables S3 and S4), as previously reported in other papers (Rauser and Meuwly 1995; Souza and Rauser 2003; Nocito et al. 2011). Moreover, fractioning of metals accumulated in the root also revealed that the three main fractions were in a dynamic equilibrium in which the increases in Zn or Cd external concentration, in the presence of a steady amount of Cd or Zn, respectively, resulted in changes in the amounts of the two metal ions in each fraction (Additional file 1: Tables S1 and S2).

Focusing the attention on the cationic fractions (Table 1, Additional file 1: Tables S1 and S2) we can make some educated guesses about changes in the relative mobility of Zn and Cd into the root, since, as discussed above, only the metal ions belonging to these fractions have all the requisites to be considered potentially mobile and then available for root-to-shoot translocation (Nocito et al. 2011; Additional file 1: Figure S2). In fact, the fraction of buffer-soluble Zn and Cd not immobilized by the anion exchanger may have come from Zn and Cd ions in free cytosolic form or bound with organic acids or NA to form cellular complexes of relatively high mobility inside the plant (Krotz et al. 1989; Deinlein et al. 2012).

**Fig. 5:**
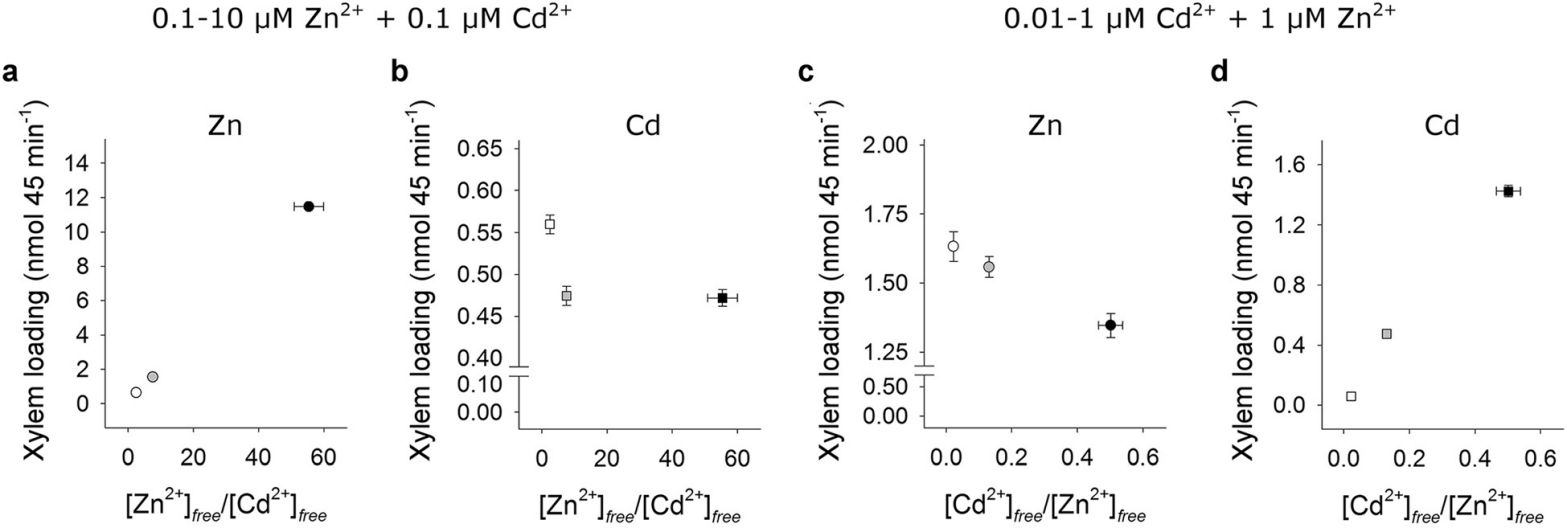
Analysis of metal xylem loading as a function of Zn and Cd concentration in the mobile fractions. Rice plants were hydroponically grown and exposed for a 10-day period to increasing Zn external concentrations (from 0.1 to 10 μM), in the presence of 0.1 μM Cd^2+^ (**a** and **b**), or to different Cd concentrations (from 0.01 to 1 μM) in the presence of 1 μM Zn^2+^ (**c** and **d**). **a** and **b** Zn (circles) and Cd (square) translocation in plants exposed to 0.1 (*white*), 1 (*grey*) and 10 (*black*) μM Zn^2+^, in the presence of 0.1 μM Cd^2+^. **c** and **d** Zn (circles) and Cd (square) translocation in plants exposed to 0.01 (*white*), 0.1 (*grey*) and 1 (*black*) μM Cd^2+^, in the presence of 1 μM Zn^2+^. Data reported in figures derive from Fig. 2 and Additional file 1: Tables S1 and S2

Increases in Zn external concentration, in the presence of a steady amount of Cd, did not produced significant changes in the amount of Cd ions in the mobile fractions but conversely enhanced the amount of free Zn ions in the same fractions. Such behaviors may be ascribable to competition phenomena between the two ions for both root absorption and negative charges on cellular matrices or apoplast components. In fact, the increases in Zn external concentration not only resulted in a reduced total amount of Cd in the roots, but also displaced non-mobile Cd ions from cellular matrices (Table 1, Additional file 1: Tables S1 and S2). On the other hand, the increases in Cd external concentration, in the presence of a steady amount of Zn, significantly enhanced the amount of both Zn and Cd ions in the mobile fractions (Table 1, Additional file 1: Tables S1 and S2). Also in this case, we can speculate that the gradual saturation of cellular matrices with Cd ions may have displaced Zn ions leading to a transient increase in the activity of the free Zn forms that, in turn, was only partially counterbalanced by a weak increase in the amount of Zn ions immobilized with thiol compounds in the LMW complexes (Additional file 1: Figure S3, Tables S3 and S4). Finally, by plotting the xylem loading data obtained in the two experimental setups as a function of the Zn/Cd or Cd/Zn concentration ratios in the mobile fractions, we can easily deduce that increases in the Zn/Cd ratio did not produce fully reciprocal effects on Zn and Cd translocation, whilst increases in the Cd/Zn ratio resulted in fully reciprocal effects (Fig. 5). Such a finding strongly confirms the hypothesis that Cd ions may use at least two distinct pathways to be translocated from roots to shoots. The first one – shared with Zn – is probably used for Zn translocation in physiological conditions, whilst the second one appears as a Zn-insensitive route that Cd may preferentially use when the first pathway is saturated with Zn. Moreover, the Zn-insensitive pathway we postulate seems to be independent from Cd stress or nutritional status reached by plants under Zn excess (10 μM), since the partial inhibitory effect exerted by Zn on Cd translocation was also observed in short-term PETIS experiments performed with unstressed rice plants (Fig. 4). In fact, time-course analysis of Cd systemic movement in the plants revealed that: i) the rate of Cd translocation was significantly reduced by changing the external concentration of Zn from 0.1 to 1 μM and then remained unaffected following a further increase in Zn availability (Fig. 4d); ii) the rates of Cd translocation measured in the presence of different Zn external concentrations significantly differed just starting from the first hour of exposure (Fig. 4d), suggesting that the hypothetical Zn-insensitive pathway was constitutively expressed and not induced by changes in the nutritional status of the rice plants. It is also noteworthy that rates of Cd translocation measured in the presence of 1 and 10 μM Zn^2+^ did not significantly differ even if the highest Zn concentration we tested strongly reduced the rate of Cd accumulation in the roots (Fig. 4). Such a finding clearly confirms that the gradual saturation of the Zn-dependent pathway with Zn may force Cd ions to move through the second pathway, suggesting that the two translocation routes normally compete for free Cd ions into the root. Moreover, our main conclusion seems to be further supported by the paper of Satoh-Nagasawa et al. (2012) which showed that rice mutants defective for OsHMA2 – the main candidate transport system so far described in rice as involved in xylem-mediated Zn^2+^/Cd^2+^ translocation (Nocito et al. 2011; Satoh-Nagasawa et al. 2012; Takahashi et al. 2012; Satoh-Nagasawa et al. 2013; Yamaji et al. 2013) – had a residual capacity to translocate Cd to the shoots. Finally, the fact that wild-type plants - rather than mutants or genetically manipulated plants - were used in the present study strengthens the notion that long-distance transport of Cd along alternative pathways is a physiological process that generally occurs under Zn excess and does not result from anomalous or compensatory expression of other non-selective transporters.

## Conclusions

Our data provide several forms of evidence to support the hypothesis that at least two distinct and competing pathways may take part in mediating root-to-shoot Cd translocation in rice. The first one, prevailing at relatively low Zn concentrations, could involve OsHMA2 as the Zn^2+^/Cd^2+^ xylem loading system, while the second one appears to involve a Zn-insensitive system that still needs to be identified among transporters showing Cd-efflux activity in the vascular tissues of the roots.

## Methods

### Plant Material, Growth Conditions and Sampling

Rice (*O. sativa* L. cv. Roma) caryopses were placed on filter paper saturated with distilled water and incubated in the dark at 26 °C. Seven days later, seedlings were transplanted into 5 L plastic tanks (eight seedlings per tank) containing the following complete nutrient solution (pre-growing solution): 1.5 mM KNO_3_, 1 mM Ca(NO_3_)_2_, 500 μM MgSO_4_, 250 μM NH_4_H_2_PO_4_, 25 μM Fe-tartrate, 46 μM H_3_BO_3_, 9 μM MnCl_2_, 1 μM ZnCl_2_, 0.3 μM CuCl_2_, 0.1 μM (NH_4_)_6_Mo_7_O_24_, 30 μM Na_2_O_3_Si (pH 6.5). Seedlings were kept for a 12-day pre-growing period in a growth chamber maintained at 26 °C and 80 % relative humidity during the 16 h light period and at 22 °C, and 70 % relative humidity during the 8 h dark period. Photosynthetic photon flux density was 400 μmol m^−2^ s^−1^. At the end of the pre-growing period, plants were differentially exposed for a 10-day period to different concentrations of Zn (0.1, 1 and 10 μM), in the absence or presence of a steady amount of Cd (0.1 μM), or to different concentrations of Cd (0, 0.01, 0.1 and 1 μM), in the presence of a steady amount of Zn (1 μM), by supplementing the pre-growing solution with different amounts of ZnCl_2_ and/or CdCl_2_. All hydroponic solutions were renewed daily to minimize nutrient depletion. Plants were harvested and roots were washed for 10 min in ice-cold 5 mM CaCl_2_ solution to displace extracellular Cd (Rauser 1987), rinsed in distilled water and gently blotted with paper towels. Shoots were separated from roots and the tissues were frozen in liquid N_2_ and stored at −80 °C, or analyzed immediately.

For positron-emitting tracer imaging system (PETIS) experiments, rice (*O. sativa* L. cv. Nipponbare) caryopses were germinated on a plastic mesh floating on distilled water, in the dark at 25 °C. Seven days later, seedlings were pre-grown in a one-quarter-strength Kimura B nutrient solution for 7 days and then grown in a full-strength Kimura B nutrient solution for another 7-day period. The Kimura B nutrition solution contained 0.70 mM (NH_4_)_2_SO_4_, 0.17 mM Na_2_HPO_4_, 0.27 mM K_2_SO_4_, 0.47 mM MgSO_4_ · 7H_2_O, 0.37 mM CaCl_2_, 11 mg L^−1^ FeC_6_H_5_O_7_ (Fe citrate), 0.16 μM CuSO_4_, 0.15 μM ZnSO_4_ · 7H_2_O, 0.10 μM Na_2_MoO_4_ · 2H_2_O, 15 μM H_3_BO_3_, and 4.6 μM MnSO_4_ · 4H_2_O (pH 5.5). Plants were kept in a growth chamber maintained at 30 °C and 65 % relative humidity during the 16 h light period and at 25 °C and 65 % relative humidity during the 8 h dark period. Photosynthetic photon flux density was 400 μmol m^−2^ s^−1^.

### Determination of Zn and Cd in Roots and Shoots

Samples of 200 mg fresh weight (FW) were mineralized at 120 °C in 5 mL 14.4 M HNO_3_, clarified with 1.5 mL 33 % (w/v) H_2_O_2_, and finally dried at 80 °C. The mineralized material was dissolved in 5 mL 0.1 M HNO_3_ and filtered on a 0.45 μm nylon membrane. Zn and Cd content was measured by inductively coupled plasma mass spectrometry (ICP-MS; Bruker Aurora M90 ICP-MS).

### Analysis of Root-to-Shoot Zn and Cd Translocation

At the end of the exposure period, shoots were cut at 2 cm above the roots with a microtome blade. Xylem sap exuded from the lower cut surface was collected for 45 min and stored into 1.5 mL plastic vials. The amount of collected sap was determined by weighing and the concentration of Zn and Cd was measured by ICP-MS.

### Determination of Total non-Protein Thiols

Roots were pulverized using mortar and pestle in liquid N_2_ and stored frozen in a cryogenic tank. Four hundred milligrams of root powders were extracted in 600 μL of 1 M NaOH and 1 mg mL^−1^ NaBH_4_, and the homogenate was centrifuged for 15 min at 13 000 *g* and 4 °C. Four hundred microliters of supernatant were collected, 66 μL of 37 % HCl was added and then the mixture was centrifuged again for 10 min at 13 000 *g* and 4 °C. For the quantification, volumes of 200 μL of the supernatant were collected and mixed with 800 μL of 1 M K-Pi buffer (pH 7.5) containing or not 0.6 mM Ellman’s reagent {[5,5′-dithiobis(2-nitrobenzoic acid); DTNB]}. The samples’ absorbances at 412 nm were then spectrophotometrically measured.

### Zn and Cd Fractioning

Metal fractioning was carried out essentially as described by Rauser and Meuwly (1995). Briefly, frozen root tissues (2 g FW) were pulverized in a cold mortar with a pestle and then homogenized with ice-cold N_2_-purged 100 mM Tris–HCl (pH 8.6), 1 mM phenylmethanesulfonyl fluoride (PMSF) and 1 % (v/v) Tween 20 at the ratio of 1 mL of buffer to 1 g tissue FW. The homogenate was centrifuged at 4 °C and 48 000 *g* for 6 min, the supernatant (extract 1) was collected and frozen immediately in liquid N_2_, and the pellet was resuspended in a volume of N_2_-purged 10 mM Tris–HCl (pH 8.6) and 1 % (v/v) Tween 20, previously used to rinse the mortar kept on ice. The suspension was centrifuged again, and the supernatant (extract 2) was collected and added to the extract 1 for freezing. Resuspension and centrifugation of the homogenized tissue debris was repeated four more times to collect extracts 3–6. At the end of this sequence, the pellet was suspended in a volume of ice-cold 100 mM HCl, centrifuged at 4 °C and 48 000 *g* for 6 min and the supernatant (extract 7) was retained. This sequence was repeated two more times to obtain extracts 8 and 9. The exhausted pellet was transferred to a glass tube, mineralized at 120 °C in 10 mL 14.4 M HNO_3_, clarified with 3 mL 33 % (w/v) H_2_O_2_ and finally dried at 80 °C. The mineralized material was dissolved in 5 mL 0.1 M HNO_3_ and filtered on a 0.45 μm nylon membrane.

Extracts 1 to 6 were resolved into two fractions, referred to as anionic and cationic, by anion-exchange chromatography. Buffer extract was loaded, at 20 mL h^−1^, onto a 0.5 × 2 cm column of diethylaminoethyl cellulose (DEAE) Sephadex A-25 (GE Healthcare) equilibrated with 10 mM Tris–HCl (pH 8.6). After loading, the column was washed with 50 mL of equilibrating buffer to remove unadsorbed solutes. All the fluid passing through the anion exchanger was collected for Zn and Cd analysis (cationic fraction). Anionic material was eluted with 6 mL of 10 mM Hepes (pH 8.0) and 1 M KCl. Five milliliters of the anionic fraction so obtained were further resolved by gel filtration on a Sephadex G-50 (Sigma) column (0.8 × 130 cm) equilibrated with 10 mM Hepes (pH 8.0) and 300 mM KCl. The column was developed in equilibrating buffer at 12.5 mL h^−1^ at 4 °C. The absorbance at 254 nm was recorded and fractions of about 5 mL were collected for Zn, Cd, and NPT analysis. The column was calibrated by using 5 mL of 0.25 % (w/v) blue dextran 2000 and 1 % (w/v) K_3_Fe(CN)_6_ to estimate void (*V*_0_) and total volume (*V*_t_), respectively. The partition coefficient, *K*_av_, was calculated using the following equation: *K*_av_ = (*V*_e_ - *V*_0_)/(*V*_t_ - *V*_0_), where *V*_e_ was the elution volume. For total non-protein thiol determination, selected fractions from gel filtration were pooled in a glass tube, lyophilized, and finally analyzed as described above. The amount of Zn and Cd ions in mineralized pellets, extracts and column effluents was measured by ICP-MS.

### ^107^Cd Tracer

^107^Cd was produced as described by Fujimaki et al. (2010). Briefly, a silver foil was bombarded for 2 h with a 17-MeV energetic proton beam, at a current of 5 μA from a cyclotron, at Takasaki Ion Accelerators for Advanced Radiation Application, Japan Atomic Energy Agency. The irradiated material was dissolved in HNO_3_, and then diluted in warm water. Silver was precipitated by adding to the solution a 0 to 2 M gradient of HCl. Supernatant, containing ^107^Cd, was filtered, dried and dissolved in water. Aliquots of ^107^Cd (6.7 MBq) were added to 12.2 mL of the culture solutions [0.5 mM CaCl_2_, 0.1 μM nonradioactive CdCl_2_, different concentrations (0.1, 1, 10 μM) of ZnSO_4_] used for each plant in the experiments.

### PETIS Imaging

Rice plants were transferred into appropriate 120 × 14 × 10 mm plastic vessels, containing 12 mL of full-strength Kimura B nutrient solution. Before starting the experiments, plants were acclimatized for 1 h in aerated 0.5 mM CaCl_2_ solutions. In a typical experiment, 6 vessels – each containing one plant – were placed in the mid-plane between two opposing detector heads of the PETIS apparatus (a modified type of PPIS-4800; Hamamatsu Photonics, Japan). Detectors were focused on the plants in order to observe the culture solutions, the whole root apparatus, the shoot bases, and the proximal portions of the shoots, in a 12 × 19 cm field of view (FOV). The entire setup was installed in a growth chamber maintained at 30 °C and 65 % relative humidity in continuous light at the density of 400 μmol m^−2^ s^−1^.

PETIS experiments were started by injecting the ^107^Cd marked culture solutions in the different plastic vessels. All the solutions were continuously stirred with gentle aeration in order to maintain a uniform composition. The surface level of the solution in the vessels was maintained by supplying fresh 0.5 mM CaCl_2_ solutions with an appropriate solution supply system. Images of the ^107^Cd distribution in the FOV were obtained every one minute for 24 h. The data of serial images obtained from the PETIS apparatus were analyzed for ^107^Cd distribution in specific regions of interest (ROIs; background, culture solution, distal roots, proximal roots, shoot base, and proximal shoot) using NIH ImageJ 1.45 s software (Schneider et al. 2012). ROIs were manually selected on the image data and the time-activity curves (time-courses of signal intensity in the ROIs) were generated and used to estimate the dynamics of Cd in the culture solution, whole root apparatus, shoot base, and proximal shoot, as described by Yoshihara et al. (2014).

### Autoradiography

At the end of the PETIS experiments, plants were dissected, fixed on paper sheets with adhesive tape, and then placed in contact with imaging plates (BAS-MS2040, GE Healthcare, Japan) in cassettes for 3 days. The imaging plates were scanned using a Bio Imaging Analyzer (Typhoon FLA 7000, GE Healthcare, Japan) to generate the autoradiographic images of ^109^Cd in the plants. In fact, ^109^Cd with a longer half-life (461 days) than ^107^Cd (6.5 h) was also obtained at a minor ratio (approximately 1:3000) in the production process of ^107^Cd.

### Statistical Analysis

Statistical analysis was carried out using SigmaPlot for Windows version 11.0 (Systat Software, Inc., Chicago, IL, USA). Quantitative values are presented as mean ± standard error of the mean (SE). Significance values were adjusted for multiple comparisons using the Bonferroni correction. Statistical significance was at *P* < 0.05. Student’s *t*-test was used to assess the significance of the observed differences between plants exposed or not to Cd in each Zn exposure condition analyzed. Statistical significance was at *P* ≤ 0.001.

## Additional File

Additional file 1: Table S1. Fractioning of Zn ions retained in root. **Table S2** Fractioning of Cd ions retained in root. **Table S3** Zn ions retained in the root in complexes with thiols. **Table S4** Cd ions retained in the root in complexes with thiols. **Figure S1** Relationship between Zn and Cd ions loaded in the xylem sap and Zn and Cd concentration in shoots. **Figure S2** Fractioning of Zn and Cd ions in the buffer extract. **Figure S3** Zn‐ and Cd‐binding complexes resolved by gel filtration chromatography. **Figure S4** Autoradiography and time‐course analysis of Cd accumulation in the shoot base. **Figure S5** Another set of time‐course analysis of Cd systemic movement. (PDF 1.39 mb)

## Abbreviations

Cd: cadmium
DEAE: diethylaminoethyl cellulose
DTNB: 5,5′-dithiobis(2-nitrobenzoic acid)
FOV: field of view
HMW: high-molecular-weight
ICP-MS: inductively coupled plasma mass spectrometry
LMW: low-molecular-weight
NPTs: non-protein thiols
PCs: phytochelatins
PETIS: positron-emitting tracer imaging system
ROIs: regions of interest
RT-PCR: reverse transcription polymerase chain reaction
Zn: zinc
