# Supplementary material for: Kinetic Analysis of Zinc/Cadmium Reciprocal Competitions Suggests a Possible Zn-Insensitive Pathway for Root-to-Shoot Cadmium Translocation in Rice
Source: Rice (N Y). 2016 Apr 12;9:16. doi: 10.1186/s12284-016-0088-3 (PMC4828370; doi:10.1186/s12284-016-0088-3)
Supplement: Additional file 1: Table S1. — Fractioning of Zn ions retained in root. Table S2 Fractioning of Cd ions retained in root. Table S3 Zn ions retained in the root in complexes with thiols. Table S4 Cd ions retained in the root in complexes with thiols. Figure S1 Relationship between Zn and Cd ions loaded in the xylem sap and Zn and Cd concentration in shoots. Figure S2 Fractioning of Zn and Cd ions in the buffer extract. Figure S3 Zn‐ and Cd‐binding complexes resolved by gel filtration chromatography. Figure S4 Autoradiography and time‐course analysis of Cd accumulation in the shoot base. Figure S5 Another set of time‐course analysis of Cd systemic movement. (PDF 1.39 mb) [file 12284_2016_88_MOESM1_ESM.pdf]

Table S1. Fractioning of Zn ions retained in root.

Rice plants were hydroponically grown and exposed for a 10-day period to increasing Zn external concentrations (from 0.1 to 10 µM), in the absence or presence of 0.1 µM Cd^{2+}, or to different Cd concentrations (from 0 to 1 µM) in the presence of 1 µM Zn^{2+}. Zn retained by roots was extracted with buffer and acid using the sequential procedure described in Materials and Methods section. Data are means and SE of three experiments, each performed with eight plants (n = 3). Different letters indicate significant differences between treatments ($P < 0.05$). DW, dry weight.

Metal concentration		Zn content				
Zn	Cd	Buffer soluble (1-6)		Acid soluble (7-9)	Ash	Total
		Anionic	Cationic			
µM				*nmol g^{-1} DW*		
0.1	0	42.05 ± 1.39 (a)	143.03 ± 3.86 (a)	240.95 ± 7.71 (a)	51.78 ± 1.91 (a)	477.81 ± 14.88 (a)
1	0	60.00 ± 1.56 (a)	420.00 ± 14.70 (a)	746.00 ± 22.38 (a)	121.00 ± 4.60 (a)	1347.00 ± 43.24 (b)
10	0	384.04 ± 13.44 (b)	3136.82 ± 128.61 (b)	13634.40 ± 545.38 (b)	618.49 ± 25.36 (b)	17773.74 ± 712.78 (c)
0.1	0.1	45.37 ± 1.72 (a)	206.02 ± 6.59 (a)	200.67 ± 7.22 (a)	46.79 ± 1.50 (a)	498.85 ± 17.04 (a)
1	0.1	110.00 ± 3.85 (b)	632.25 ± 22.13 (a)	475.01 ± 19.95 (a)	92.21 ± 3.13 (a)	1309.46 ± 49.06 (b)
10	0.1	534.96 ± 21.93 (c)	4541.08 ± 177.10 (b)	11546.99 ± 288.67 (b)	587.44 ± 25.26 (b)	17210.47 ± 512.97 (c)
1	0	60.00 ± 1.56 (a)	420.00 ± 14.70 (a)	746.00 ± 22.38 (a)	121.00 ± 4.60 (a)	1347.00 ± 43.24 (a)
1	0.01	95.59 ± 2.87 (b)	486.91 ± 17.04 (a)	620.59 ± 21.10 (b)	113.54 ± 3.29 (a)	1316.63 ± 44.30 (a)
1	0.1	110.00 ± 3.85 (b)	632.25 ± 22.13 (b)	475.01 ± 19.95 (c)	92.21 ± 3.13 (b)	1309.46 ± 49.06 (a)
1	1	145.12 ± 4.50 (c)	696.00 ± 24.36 (b)	364.00 ± 10.56 (d)	82.03 ± 3.12 (b)	1287.16 ± 42.53 (a)

Table S2. Fractioning of Cd ions retained in root.

Rice plants were hydroponically grown and exposed for a 10-day period to increasing Zn external concentrations (from 0.1 to 10 µM) in the presence of 0.1 µM Cd^{2+}, or to different Cd concentrations (from 0 to 1 µM) in the presence of 1 µM Zn^{2+}. Cd retained by roots was extracted with buffer and acid using the sequential procedure described in Materials and Methods section. Data are means and SE of three experiments, each performed with eight plants (n = 3). Different letters indicate significant differences between treatments ($P < 0.05$). ND, not detectable; DW, dry weight.

Metal concentration		Cd content				
Zn	Cd	Buffer soluble (1-6)		Acid soluble (7-9)	Ash	Total
		Anionic	Cationic			
µM				*nmol g^{-1} DW*		
0.1	0.1	140.10 ± 5.04 (a)	81.66 ± 2.61 (a)	608.83 ± 21.31 (a)	149.79 ± 5.69 (a)	980.36 ± 34.66 (a)
1	0.1	84.57 ± 3.55 (b)	83.00 ± 2.82 (a)	444.00 ± 14.21 (b)	84.00 ± 2.94 (b)	695.57 ± 23.52 (b)
10	0.1	72.76 ± 1.82 (b)	82.00 ± 3.53 (a)	397.07 ± 15.88 (b)	83.03 ± 3.40 (b)	634.86 ± 24.63 (b)
1	0	ND	ND	ND	ND	ND
1	0.01	5.85 ± 0.20 (a)	11.06 ± 0.32 (a)	62.79 ± 2.20 (a)	11.99 ± 0.36 (a)	91.69 ± 3.08 (a)
1	0.1	84.57 ± 3.55 (b)	83.00 ± 2.82 (b)	444.00 ± 14.21 (b)	84.00 ± 2.94 (b)	695.57 ± 23.52 (b)
1	1	624.33 ± 18.11 (c)	349.49 ± 13.28 (c)	3442.60 ± 92.95 (c)	845.28 ± 26.20 (c)	5261.71 ± 150.54 (c)

Table S3. Zn ions retained in the root in complexes with thiols.

Rice plants were hydroponically grown and exposed for a 10-day period to increasing Zn external concentrations (from 0.1 to 10 µM), in the absence or presence of 0.1 µM Cd^{2+}, or to different Cd concentrations (from 0 to 1 µM) in the presence of 1 µM Zn^{2+}. The anionic fraction from buffer extracts 1–6 was chromatographed on a Sephadex G-50 column. Zn eluted was measured by ICP-MS. Data are means and SE of three experiments, each performed with eight plants (n = 3). Different letters indicate significant differences between treatments ($P < 0.05$). LMW, low molecular weight Zn-binding complexes; HMW, high molecular weight Zn-binding complexes. ND, not detectable; DW, dry weight.

Metal concentration		Zn		
Zn	Cd	HMW	LMW	Complexed with thiols
µM			*nmol g^{-1} DW*	
0.1	0	ND	21.74 ± 0.70 (a)	21.74 ± 0.70 (a)
1	0	ND	47.62 ± 1.76 (a)	47.62 ± 1.76 (a)
10	0	ND	343.45 ± 14.42 (b)	343.45 ± 14.42 (b)
0.1	0.1	ND	24.36 ± 0.83 (a)	24.36 ± 0.83 (a)
1	0.1	ND	84.75 ± 2.97 (b)	84.75 ± 2.97 (b)
10	0.1	ND	507.87 ± 19.61 (c)	507.87 ± 19.61 (c)
1	0	ND	47.62 ± 1.76 (a)	47.62 ± 1.76 (a)
1	0.01	ND	72.89 ± 2.70 (b)	72.89 ± 2.70 (b)
1	0.1	ND	84.75 ± 2.97 (b)	84.75 ± 2.97 (b)
1	1	ND	121.21 ± 4.97 (c)	121.21 ± 4.97 (c)

Table S4. Cd ions retained in the root in complexes with thiols.

Rice plants were hydroponically grown and exposed for a 10-day period to increasing Zn external concentrations (from 0.1 to 10 µM), in the absence or presence of 0.1 µM Cd^{2+}, or to different Cd concentrations (from 0 to 1 µM) in the presence of 1 µM Zn^{2+}. The anionic fraction from buffer extracts 1–6 was chromatographed on a Sephadex G-50 column. Cd eluted was measured by ICP-MS. Data are means and SE of three experiments, each performed with eight plants (*n* = 3). Different letters indicate significant differences between treatments ($P < 0.05$). LMW, low molecular weight Cd-binding complexes; HMW, high molecular weight Cd-binding complexes. ND, not detectable; DW, dry weight.

Metal concentration		Cd		
Zn	Cd	HMW	LMW	Complexed with thiols
µM			*nmol g⁻¹ DW*	
0.1	0.1	96.89 ± 3.00 (a)	34.18 ± 1.13 (a)	131.07 ± 4.13 (a)
1	0.1	45.78 ± 1.51 (b)	31.83 ± 1.18 (a)	77.61 ± 2.69 (b)
10	0.1	33.82 ± 1.29 (c)	33.19 ± 1.33 (a)	67.00 ± 2.61 (b)
1	0	ND	ND	ND
1	0.01	ND	5.00 ± 0.17 (a)	5.00 ± 0.17 (a)
1	0.1	45.78 ± 1.51 (a)	31.83 ± 1.18 (b)	77.61 ± 2.69 (b)
1	1	473.66 ± 17.05 (b)	134.35 ± 5.24 (c)	608.01 ± 22.29 (c)

Figure S1

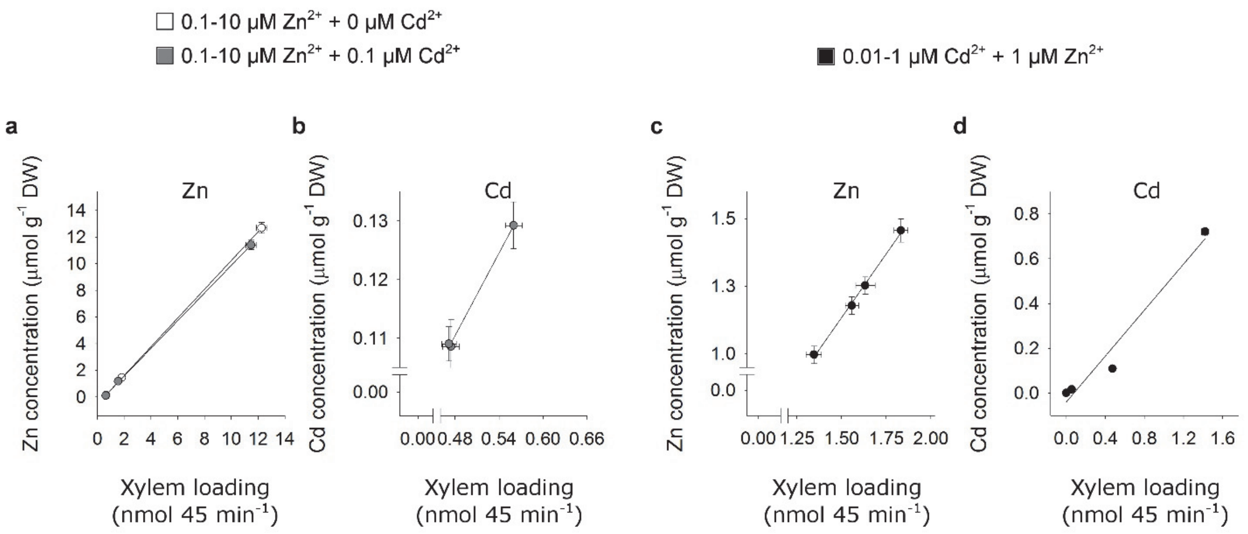

Relationship between Zn and Cd ions loaded in the xylem sap and Zn and Cd concentration in shoots. Rice plants were hydroponically grown and exposed for a 10-day period to increasing Zn external concentrations (from 0.1 to 10 µM), in the absence or presence of 0.1 µM Cd^{2+} (**a** and **b**), or to different Cd concentrations (from 0 to 1 µM) in the presence of 1 µM Zn^{2+} (**c** and **d**). **a** Zn xylem loading vs Zn shoot concentration in the absence (white circles) and in the presence (grey circles) of a steady amount of Cd. **b** Cd xylem loading vs Cd shoot concentration in the presence of a steady amount of Cd. **c** Zn xylem loading vs Zn shoot concentration in the presence of a steady amount of Zn. **d** Cd xylem loading vs Cd shoot concentration in the presence of a steady amount of Zn. Data are means and SE of three experiments run in triplicate (*n* = 9). DW, dry weight.

Figure S2

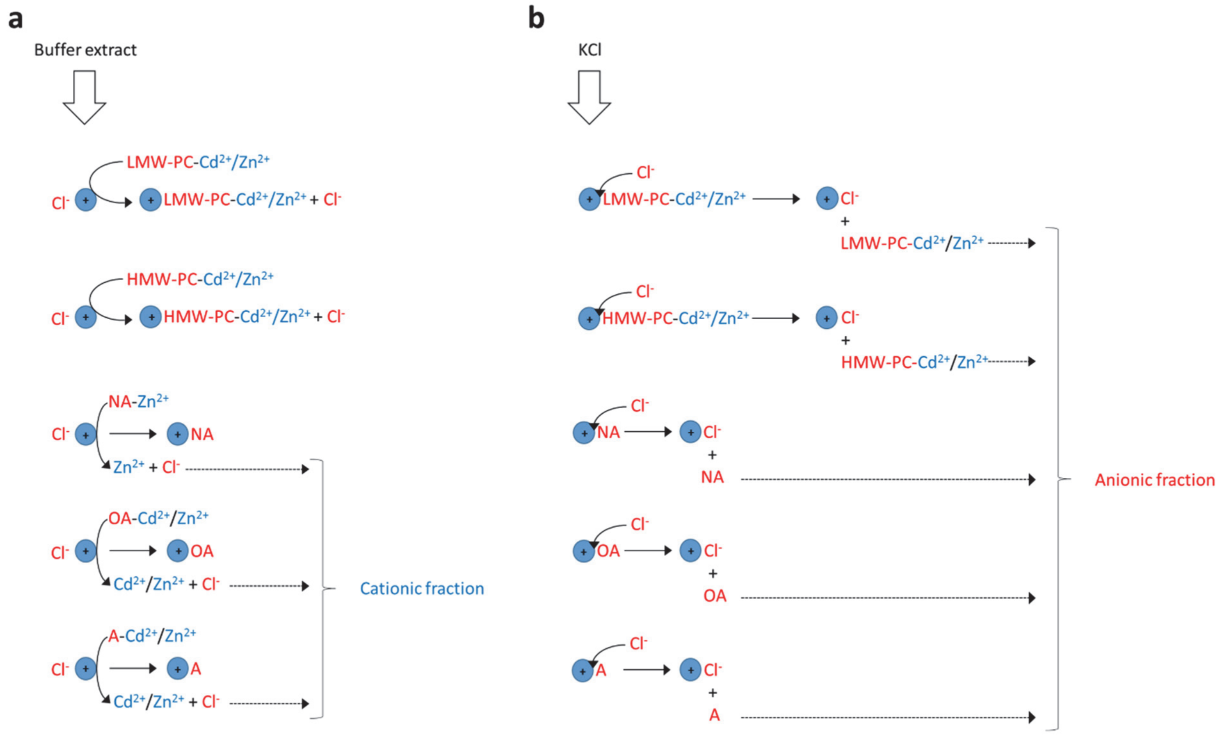

Fractioning of Zn and Cd ions in the buffer extract. **a** Buffer extract was loaded onto a column of diethylaminoethyl cellulose (DEAE) Sephadex A-25 equilibrated with 10 mM Tris-HCl (pH 8.6). During this phase LMW and HMW thiol based Zn- and/or Cd-binding complexes were adsorbed on the surface of the anion exchanger, whilst Zn and Cd ions bound with ligands (e.g. nicotianamine, organic acid or anions) to form complexes with relatively low thermodynamic stability were released in the cationic fraction. **b** Column retained anionic material was eluted with 6 mL of 10 mM Hepes (pH 8.0) and 1 M KCl to obtain an anionic fraction containing LMW and HMW thiol based Zn- and/or Cd-binding complexes. The blue color indicates cations, whilst the red one indicates anions. LMW-PC-Cd^{2+}/Zn^{2+}, LMW thiol based Zn- and/or Cd-binding complexes; HMW-PC-Cd^{2+}/Zn^{2+}, HMW thiol based Zn- and/or Cd-binding complexes; NA, nicotianamine; OA, organic acids; A, anions.

Figure S3

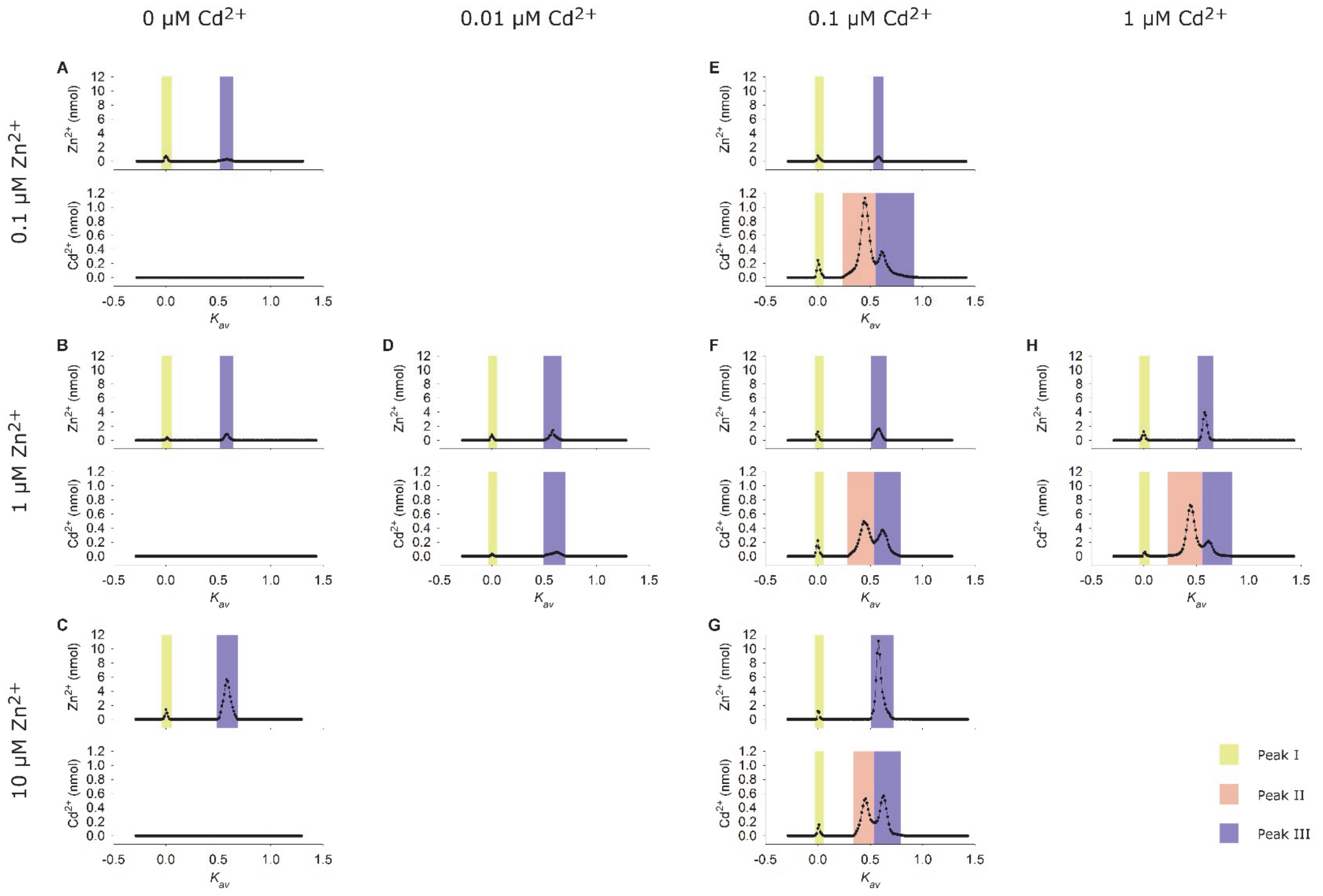

Zn- and Cd-binding complexes resolved by gel filtration chromatography. Rice plants were hydroponically grown and exposed for a 10-day period to increasing Zn external concentrations (from 0.1 to 10 µM), in the absence (**a**, **b**, **c**) or presence of 0.1 µM Cd^{2+} (**e**, **f**, **g**), or to different Cd concentrations (from 0 to 1 µM) in the presence of 1 µM Zn^{2+} (**b**, **d**, **f**, **h**). The anionic fraction from buffer extracts 1–6 was chromatographed on a Sephadex G-50 column. Zn and Cd eluted was measured by ICP-MS. Void and total volume peaks are centered at $K_{av} = 0$ and $K_{av} = 1$, respectively. Peaks I, II and III are highlighted with different colors. Data are representative of one typical experiment repeated three times with similar results.

Figure S4

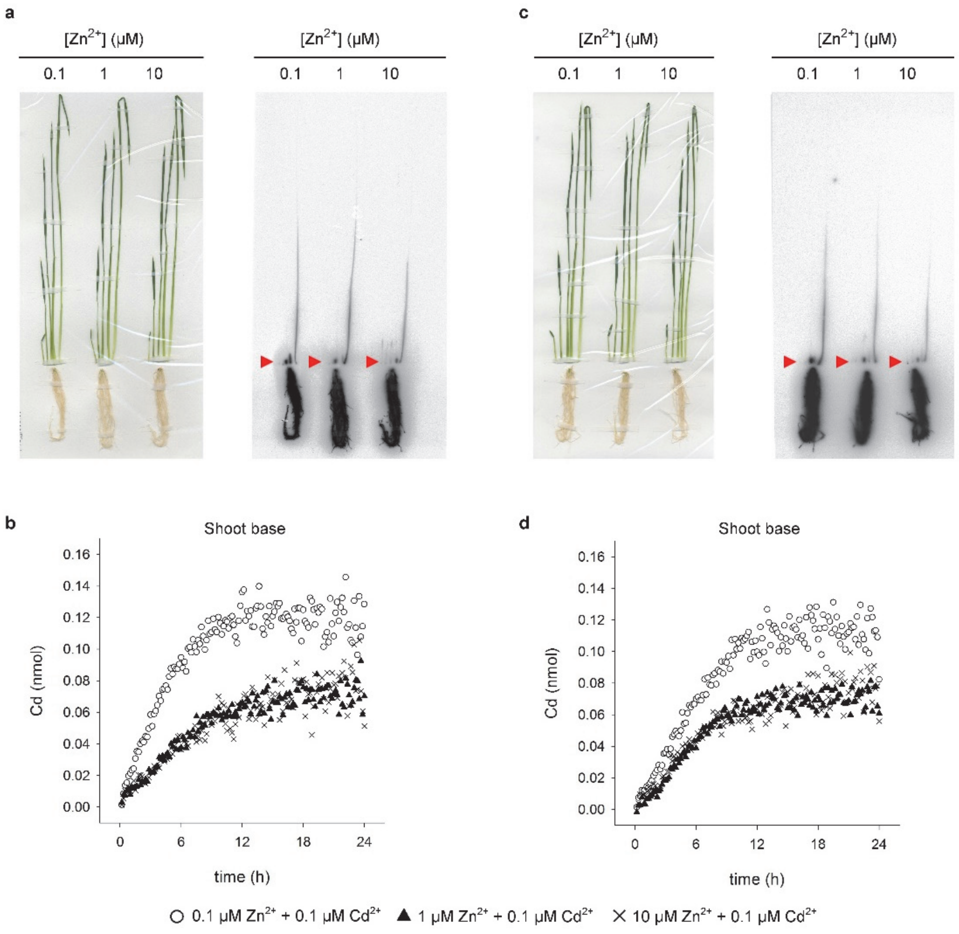

Autoradiography and time-course analysis of Cd accumulation in the shoot base. Rice plants were exposed for a 24-h period to different Zn concentrations (from 0.1 to 10 μM), in the presence of 0.1 μM Cd^{2+} enriched with ^{107}Cd. **a** and **c** Optical observation of plants at the end of the PETIS experiments (left) and the corresponding autoradiography of ^{109}Cd after three days of exposure (right). Red triangles indicate the shoot bases. **b** and **d** Time-course analysis of Cd accumulation in the shoot bases. White circles, black triangles, and thin x refer to the experiments performed in the presence of 0.1, 1 and 10 μM Zn^{2+}, respectively. Two representative set of data from two independent experiments performed with two plants for each Zn exposure condition are given.

Figure S5

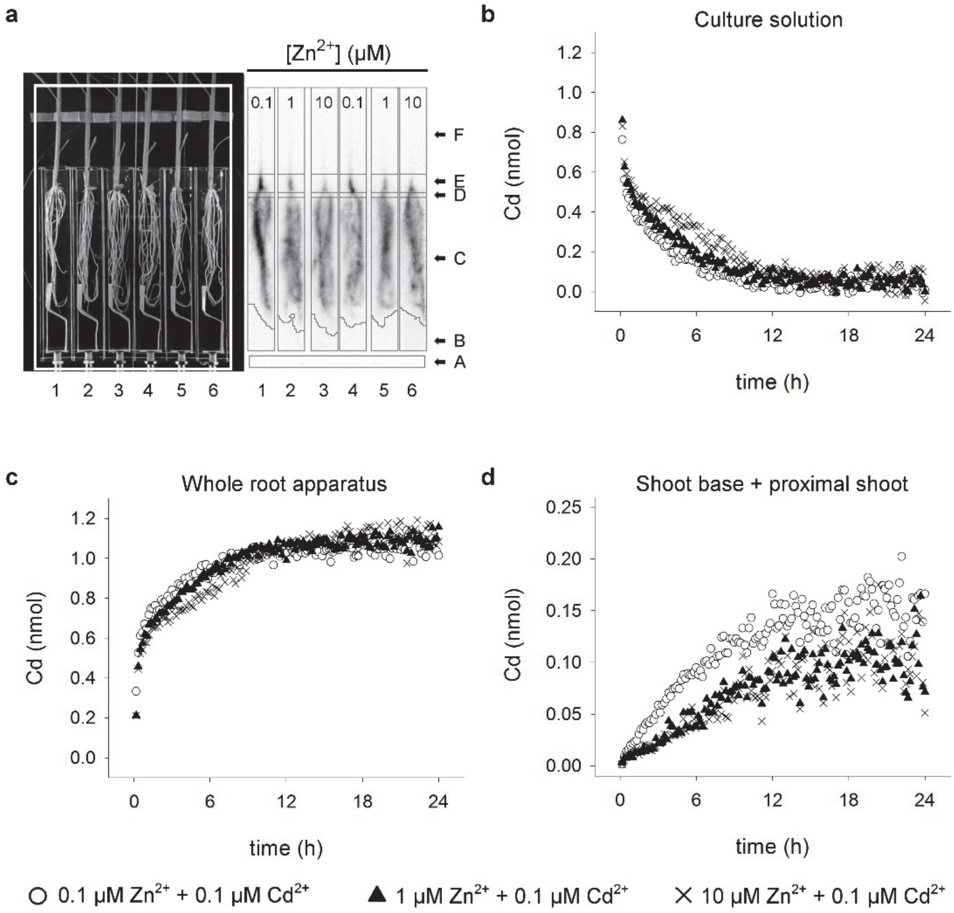

Another set of time-course analysis of Cd systemic movement. Rice plants were exposed for a 24-h period to different Zn concentrations (from 0.1 to 10 μM), in the presence of 0.1 μM Cd^{2+} enriched with ^{107}Cd. **a** Image of rice plants used in a typical PETIS experiment. The field of view of PETIS is bordered by the white continuous line. ROIs used for time-course analysis are indicated with arrows in the adjacent panel. ROI-A, background; ROI-B, culture solution; ROI-C, distal root; ROI-D, proximal root; ROI-E, shoot base; ROI-F, proximal shoot. **b**-**d**, Time-course analysis of Cd dynamics in the culture solution (**b**), whole root apparatus (**c**), and shoot base + proximal shoot (**d**). White circles, black triangles, and thin x refer to the experiments performed in the presence of 0.1 (No. 1), 1 (No. 2) and 10 (No.3) μM Zn^{2+}, respectively. A representative set of data from two independent experiments performed with two plants for each Zn exposure condition is given.
